# Quality of Animal Experiments in Anti-Angiogenic Cancer Drug Development – A Systematic Review

**DOI:** 10.1371/journal.pone.0137235

**Published:** 2015-09-30

**Authors:** Marianne Isabelle Martić-Kehl, Jannis Wernery, Gerd Folkers, Pius August Schubiger

**Affiliations:** Collegium Helveticum, ETH and University of Zurich, Zurich, Switzerland; University of Bari Medical School, ITALY

## Abstract

Translation from preclinical animal research to clinical bedside has proven to be difficult to impossible in many fields of research (e.g. acute stroke, ALS and HIV vaccination development) with oncology showing particularly low translation rates (5% vs. 20% for cardiovascular diseases). Several investigations on published preclinical animal research have revealed that apart from plain species differences, translational problems can arise from low study quality (e.g. study design) or non-representative experimental conditions (e.g. treatment schedule).

This review assessed the published experimental circumstances and quality of anti-angiogenic cancer drug development in 232 in vivo studies. The quality of study design was often insufficient; at least the information published about the experiments was not satisfactory in most cases. There was no quality improvement over time, with the exception of conflict of interest statements. This increase presumably arose mainly because journal guidelines request such statements more often recently.

Visual inspection of data and a cluster analysis confirmed a trend described in literature that low study quality tends to overestimate study outcome. It was also found that experimental outcome was more favorable when a potential drug was investigated as the main focus of a study, compared to drugs that were used as comparison interventions. We assume that this effect arises from the frequent neglect of blinding investigators towards treatment arms and refer to it as hypothesis bias.

In conclusion, the reporting and presumably also the experimental performance of animal studies in drug development for oncology suffer from similar shortcomings as other fields of research (such as stroke or ALS). We consider it necessary to enforce experimental quality and reporting that corresponds to the level of clinical studies. It seems that only clear journal guidelines or guidelines from licensing authorities, where failure to fulfill prevents publication or experimental license, can help to improve this situation.

## Introduction

The process of drug development leading to market authorization of new drugs is divided into several sub-phases, in which mechanism of action, toxicological and adverse effect studies and efficacy of an intervention are first investigated in vitro and in vivo. The most promising interventions are brought to the clinic and tested in healthy volunteers as well as in different groups of patients, assuming that the experimental results of animal research possess predictive value regarding safety and efficacy of drugs in the patient.

Statements like “virtually every achievement of the last century has depended directly or indirectly on the research with animals” [1–3] are often found in literature to emphasise the importance and necessity of animal models used in drug development and medical science. In recent years, however, there is increasing scepticism about the usefulness of animal models for the predictability of clinical efficacy [4–10]. Robert Matthews [9] has discussed the validity of the above statement in a critical article in 2008 and has concluded that it is anecdotal and does not hold true in general. He is convinced, though, and surely there is evidence that “animal models can and have provided many crucial insights that have led to major advances in medicine and surgery”. Hence, he claims that systematic investigations on the use of animal models and on the evidence that they possibly can provide are necessary.

Gill Langley, in her critical paper in 2009, has referred to the same statement as Matthews a year earlier [10]. Langley has concluded that relying on animal surrogates of human illnesses is a flawed approach in science. Her own investigations, as well as several published systematic reviews of the reliability of animal models have shown that fewer than 50% of animal studies have predicted human outcomes sufficiently. In certain fields of research, e.g. development of vaccinations against AIDS, prediction failure of chimpanzee and macaque models is 100% [10, 11].

We are convinced that animal models can be useful tools in biomedical research, but undoubtedly, it has also been observed frequently that effects found in animal models could not be translated to the clinic [9,12–17]. Furthermore, there a tendency towards overestimation of effect size for studies with low experimental quality was reported [13]. Therefore, it is important to elucidate the conditions, which must be fulfilled to get meaningful animal results for human translation.

This review tries to assess the quality of animal studies in anti-angiogenic cancer drug development. The rationale for choosing oncology comes from the particularly low translation rate in this particular field [18]. The rationale of anti-angiogenic drugs as the subject of study mainly lies in the fact that the concept of anti-angiogenic therapy was introduced in the 1980ies by Judah Folkman, who postulated the idea of starving cancers by cutting them off from blood supply. It took some time until this concept led to a research boom which started in the late 1990ies leading to several compounds tested in clinical trials, some of them were launched on the market at the beginning of this century (e.g. Sutent (Pfizer) or Avastin (Roche)). The rather restricted time period of about fifteen years of preclinical anti-angiogenic research allows an unlimited data search, which still enables an analysis of experimental quality over time but on the other hand does not produce too high inhomogeneity among the studies included in our review.

The review includes studies of animal experiments assessing the efficacy of an anti-angiogenic cancer drug. The anti-angiogenic drug can either be the “main focus” of the study or just be investigated as a comparison intervention to any other drug the study is focusing on. First, we were assessing the quality of the evaluated studies, using a checklist of ten items, the quality parameters. Second, we investigated the fulfillment of these quality criteria over time in order to analyze what would be necessary to improve the quality standards in general. The third question addressed was a potential influence of study quality on the drug efficacy determined. Fourthly, our aim was to identify clusters of studies with similar performance, quality and efficacy features.

## Methods

### Protocol and Registration

A review protocol for this work does not exist; the Cochrane Collaboration does not register systematic reviews about animal research. Nevertheless, we used the Cochrane guidelines as a basis for the planning and performance of this review.

### Eligibility Criteria

The study includes research papers and conference abstracts describing animal experiments to determine the efficacy of anti-angiogenic cancer drugs with the exception of metronomic chemotherapy. All types of outcome and study design were included. Databases were searched for English articles until 2011 (= study start).

### Information Sources

Research papers were identified via two major databases of biomedical research, PUBMED and Thomson Integrity (former Prous Science). Review articles identified through database search were screened for additional original research papers or conference abstracts by hand.

### Search

The search algorithm used for the PUBMED database is listed below: [[angiogenesis] OR [angiogenesis inhibitor]] AND [[animal model] OR [mice] OR [rat*] OR [guinea pig*] OR [dog*] OR [monkey*]] AND [preclinical].

For Thomson Integrity, the literature search was carried out within the knowledge area “Experimental Pharmacology (Subsection “Experimental Activity” or “Pharmacological Activity”)”. The search strategy for Experimental Pharmacology, subsection Experimental Activity was the following: [Experimental Activity =“Angiogenic Factors” or “Angiogenic Factors inhibition, EX VIVO” or “Angiogenic Factors inhibition, IN VITRO” or “Angiogenic Factors inhibition, IN VIVO”] AND [Material = “animal*” or “mice*” or “rat*” or”rabbit*” or “monkey*” or “dog*”]. For Experimental Pharmacology subsection Pharmacological Activity the search strategy was the following: [Pharmacological Activity = “Angiogenesis, inhibition” or “Angiogenesis (basic fibroblast growth factor-induced), inhibition” or “Angiogenesis (heparanase-induced), inhibition” or “Angiogenesis (hepatocyte growth factor-induced), inhibition” or “Angiogenesis (platelet-derived growth factor-induced), inhibition” or “Angiogenesis (sphingosine 1-phosphate-induced), inhibition” or “Angiogenesis (stem-cell factor-induced), inhibition” or “Angiogenesis (tumor necrosis factor-alpha-induced), inhibition” or “Angiogenesis (vascular endothelial growth factor-C-induced), inhibition” or “Angiogenesis (vascular endothelial growth factor-induced), inhibition” or “Angiogenesis (vascular endothelial growth factor/basic fibroblast growth factor-induced), inhibition” or “Angiogenesis, inhibition”] AND [Material = “animal*” or “mice*” or “rat*” or “hamster*” or “rabbit*” or “monkey*” or “dog*”].

### Study Selection

Studies were screened for eligibility by MIM-K and PAS independently and any review article identified through the search algorithm was hand screened for references to additional original articles and conference abstracts by PAS.

### Data Collection Process and Data Items

Data was collected independently by MIK-K. The parameters collected from studies fulfilling eligibility are listed in Tables 1 and 2. We are differentiating between parameters referring to the whole study (1) and such referring to each treatment group (2) separately.

**Table 1:** Parameters referring to each study as a whole.

Parameter Name	Description
Reference	Paper Reference
Study Number	Internal Identification Number
Publication Year	1997–2011
Type of Publication	Full article, short communication, letter, conference abstract
Purpose of Study	Screening, pre-clinical
Funding Factor	Non-industry grant, industry-founded, combination
Reasons for Model Choice	Rationale stated by the authors regarding model choice
Outcome Judgment	Judgment of the study outcome by the authors: positive, negative, neutral
Blinding (Performance, Evaluation)	Yes, no, NA
Sample Size Calculation	Yes, no, NA
Randomization (Distribution of animals to test groups)	Yes, no, NA
Allocation Concealment	Yes, no, NA
Conflict of Interest Statement	Yes, no, NA
NA = not available	

**Table 2:** Parameters referring to each treatment group.

Parameter Name	Description
Function of Intervention	Main-focus of study, comparison function within study
Outcome Measure	Tumor Size, Metastases, Survival
Species	Mouse, rat, hamster, dog
Sex	f, m, f/m, NA
Genetic Variety	Inbred, Outbred, NA
Immune-Deficiency / Co-Morbidity	T-cell-lack, B-&T-cell-lack, diabetes, none
Tumor Category	Carcinoma, sarcoma, leukemia, lymphoma & myeloma, CNS tumor, other
Model Type	Xenograft, Allograft, transgenic, spontaneous
Inoculation Type	s.c., orthotopic
Tumor Type	Primary, metastatic, both
Uniform Outcome	1 = Cure, 2 = Regression, 3 = Stable Disease, 4a = Moderate Progression, 4b = Progression, 0 = categorization not feasible (Survival), 5 = NA
Phase of Outcome Definition	Treatment, post-treatment
Adverse Effects	Yes (Details), no, NA
Error Bar Representation	NA, SEM, SD, CI(95%)
NA = not available	

**Fig 1:**
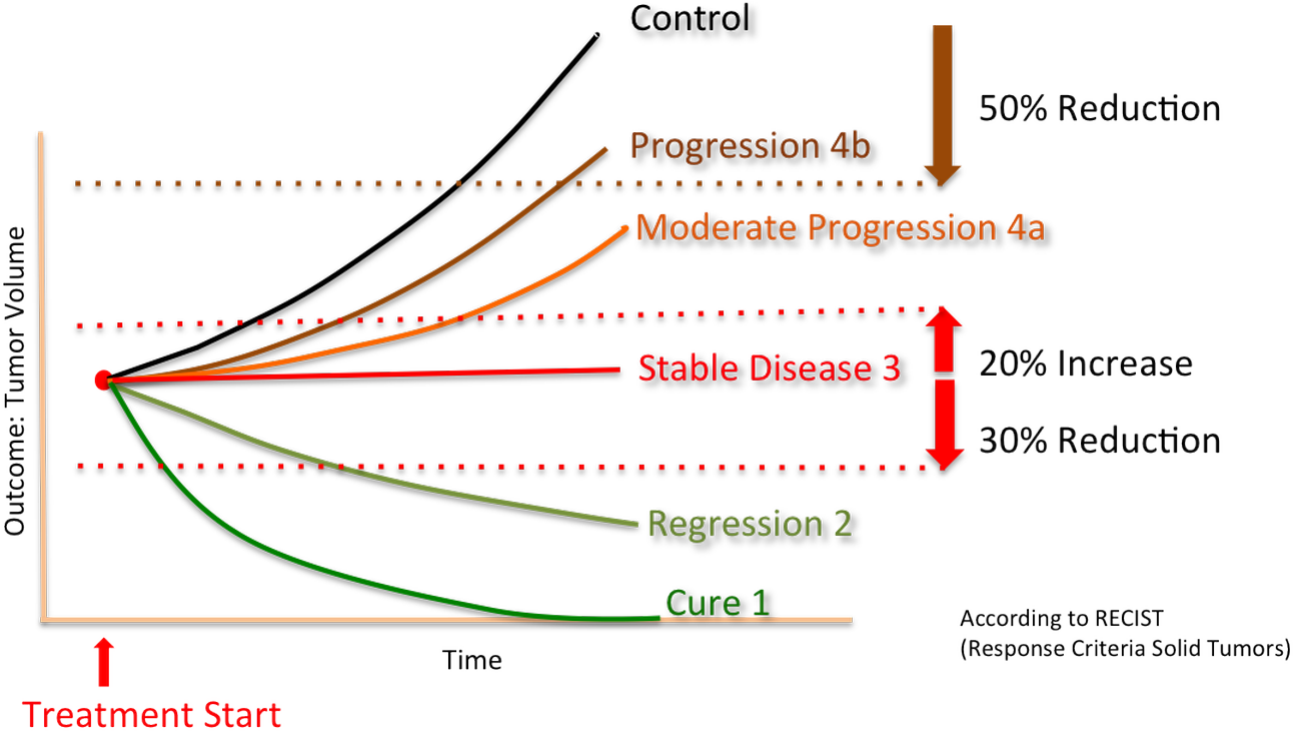
Uniform Outcome adopted from RECIST.

Due to the various types of efficacies standards (%tumor growth inhibition (TGI), ratio of tumor size between treated and control groups, time till tumor reached a certain predefined size, number of metastatses, etc) stated by the different authors, it was decided to introduce a parameter called “uniform outcome” which was adopted from the Response Criteria Solid Tumors (RECIST) defined for human solid tumors (Fig 1) [19]. This uniform outcome is a categorial variable with the following four levels; regression (level 2) was defined as a minimal reduction of 30% of the measurement parameter compared to the value at the start of treatment. Progression (level 4) was defined as a minimal increase of 20% of measurement parameter compared to the value at the start of treatment. Everything within these boundaries was considered “stable disease” (level 3). Cure (level 1) was defined as a complete absence of tumor at the time of outcome measure.

Tables 1 and 2 are describing the parameters recorded in S1 Table (http://www.collegium.ethz.ch/de/forschungsprojekte/fellowprojekt-reproduzierbarkeit/medikamentenentwicklung-aussagekraft-von-tierversuchen/Extraction Parameters–Quality of Animal Experiments in Anti-Angiogenic Cancer Drug Development). The numeric key for factor levels listed in S1 Table can be found under http://www.collegium.ethz.ch/de/forschungsprojekte/fellowprojekt-reproduzierbarkeit/medikamentenentwicklung-aussagekraft-von-tierversuchen/Numeric Key of Factor levels for extraction parameters. Additional parameters collected are listed in Table 2.

### Summary Measures and Synthesis of Results

Due to the outcome homogenization process described above, which excluded certain outcome measure completely (i.e. survival analysis), a full meta-analytic analysis was not feasible, classical summary measures like risk ratio or differences in mean did not apply.

### Additional Analyses

One main focus of this review is on the assessment of animal study quality.

Table 3 summarizes the values of study parameters we consider relevant in order to judge a preclinical cancer study as qualitatively well performed.

**Table 3:** Quality Parameters.

Quality Parameter	Beneficial Level
1. Regulatory Requirements fulfilled	Yes
2. Conflict of Interest Statement	Yes
3. Sample Size Calculation	Yes
4. Allocation Concealment	Yes
5. Randomized Allocation to Test Groups	Yes
6. Blinded Assessment of Outcome	Yes
7. Genetic Variety	Not inbred / more than one breed
8. Inoculation Type	Orthotopic
9. Tumor Type	Not exclusively primary
10. Immunodeficiency / Co-Morbidity	None

General study design parameters, which are standard in clinical trials such as blinded assessment of outcome or randomized allocation of test subjects to control and treatment groups, are represented as well as parameters regarding oncology experiments in particular.

Items 1–6 are adopted from the quality criteria checklist of Sena et al. [13]. Genetic variety: There is some evidence that proof of efficacy in a broader range of genetic background can increase external validity and robustness of results [20]. Inoculation type: Tumors growing within the stromal context of origin are reacting in a more representative way to treatment compared to tumors implanted at an “artificial” location (e.g. s.c. inoculation at well exposed body parts like the shoulder or flank). Tumor type: It has been clearly demonstrated in literature that primary tumor reaction on treatment does not necessarily correspond to metastatic behavior upon treatment [21]. Immunodeficiency / Co-morbidity: There is no evidence of the average tumor patient to suffer from typical co-morbidities or immunodeficiency.

In the first step of the data evaluation we studied the variation of the quality parameters of Table 3 over time. Specifically, it was visually checked whether the quality parameters improved over time.

In a second step, the influence of single parameters on study outcome was investigated. All treatment groups with defined uniform outcome (level 1–4) were included in this evaluation. If dose studies were performed, only the dose with the best experimental outcome was included in the evaluation. If outcome did not differ, the highest dose was chosen. First, contingency tables were generated and a Chi-Squared test was performed to assess unequal distribution of study outcome vs. particular experimental parameters. On one hand, this was performed for all available treatment groups regardless of the drug investigated in particular studies. On the other hand, studies investigating on the same drugs were investigated separately.

By summing up the number of quality criteria (Table 3) that were fulfilled, a quality score was assigned to each experiment. The fraction of experiments with uniform outcome 4 (i.e. progression) among all experiments with the same quality score was then plotted (%Outcome 4 vs. quality score) and visually inspected.

The analyses described above were pre-specified, whereas the following analysis was included during the data evaluation process: as the last step we tried to identify clusters of experiments with similar quality, study performance parameters and outcome. For this purpose, we performed a two-step cluster analysis with fixed cluster number determination. The cluster number was determined via a preliminary hierarchic cluster analysis.

### Data Analysis

Data analysis was performed using the statistics freeware RStudio (Version 0.98.1028–2009–2013 RStudio, Inc. for Mac) and R (3.0.1 GUI 1.61 Snow Leopard build 2004–2013, the R Foundation for Statistical Computing), except for cluster analysis, which was performed using the statistics software SPSS (IBM SPSS Statistics Version 22, Release 22.0.0.0 64-bit edition).

## Results

### Study Selection and Characteristics

**Fig 2:**
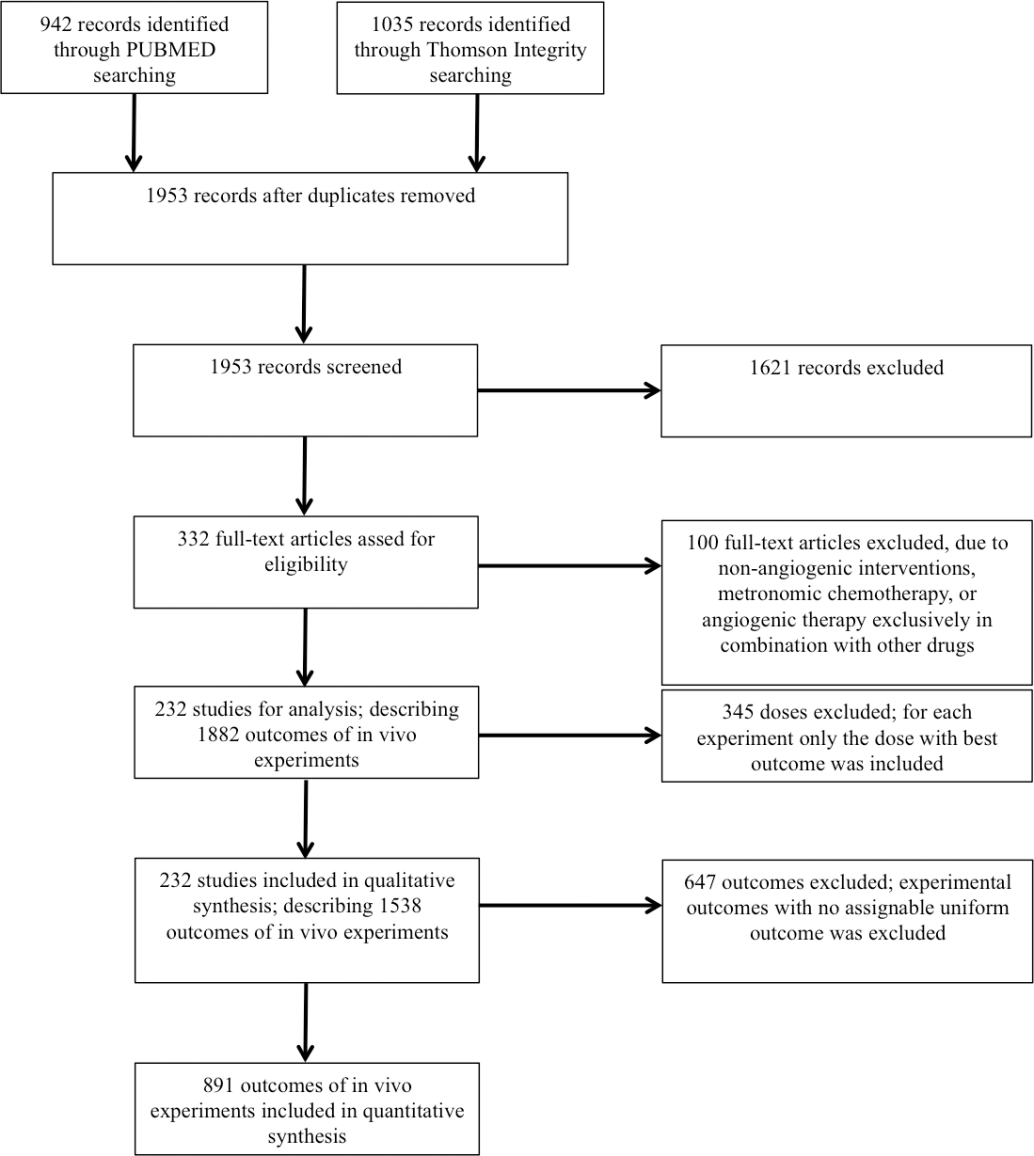
Flow chart of study selection. It is distinguished between full-text articles and individual experimental outcomes within these articles. A full-text article can include several individual experiments, each of which describing one or several anti-angiogenic drug (candidates) outcomes.

Data search identified 1953 articles in total (Fig 2).

In total we identified 232 studies: full articles (n = 196), short communications (n = 2), letters (n = 3) and conference abstracts (n = 31). In these 232 studies, a total of 299 drugs and potential drug candidates were investigated. More than half of those were mentioned in one publication only (n = 176) or two to five publications (n = 24). For the following drugs we found more than five publications each: Sutent (Pfizer, Sunitinib, SU11248, n = 17), Avastin (Roche, Bevacizumab, n = 14), Recentin (Astra Zeneca, Cediranib, AZD2171, n = 9), TNP-470 (TAP Pharmaceuticals, n = 9), Caprelsa (Astra Zeneca, Vandetanib, Zactima, ZD6474, n = 9) and Vatalanib (Bayer-Schering / Novartis, PTK787, PTK/ZK, n = 7).

Data characteristics for parameters listed in Tables 1 and 2 are described in S1 Table (http://www.collegium.ethz.ch/de/forschungsprojekte/fellowprojekt-reproduzierbarkeit/medikamentenentwicklung-aussagekraft-von-tierversuchen/Extraction Parameters–Quality of Animal Experiments in Anti-Angiogenic Cancer Drug Development). At the same place, the factor level key can be found as well (http://www.collegium.ethz.ch/de/forschungsprojekte/fellowprojekt-reproduzierbarkeit/medikamentenentwicklung-aussagekraft-von-tierversuchen/Numeric Key of Factor levels for extraction parameters).

### Data Composition and Quality Scores

91% of all drugs investigated in treatment groups with defined outcome (n_def,tot_ = 891, n_tot_ = 1538) were the main-focus of investigation of the particular study. The vast majority of animal species totally investigated were mice (96%). 1.7% of results were generated using rats. In one study (3 outcomes), golden hamsters were investigated and two articles described drugs tested in pet dogs (10 outcomes). 62% of all investigated animals groups were inbred strains and in 73%, tumors were inoculated subcutaneously (s.c.); only 15% of tumors were inoculated orthotopically and in 9% of the cases it was not reported how tumors were induced.

General quality parameters were mostly not mentioned in the studies (n_stud_ = 232). The following list shows the percentage of studies which mention the quality parameters:

- Fulfilled regulatory requirements: 47%
- Statement about potential conflict of interest: 12%
- Sample size calculation: 0.5%
- Allocation concealment: 0%
- Randomization: 41%
- Blinding: 2%

A quality score was defined by summing how many of the ten quality indicators according to Table 3 were met for each study. The highest quality score that was reached was 6 out of 10. Median score was 2.

In the studies, data variability in graphs or as numbers was either represented as standard error of the mean (SEM, 43%), as standard deviation (SD, 9%), given as individual data points or complete data range (8%), or it was not reported at all (5% of the cases). In 9% of the cases a variability representation was redundant (e.g. survival analysis) and in 26% of the cases data was not represented as numbers or graphs.

Distribution of the given study parameters changed only marginally when only full preclinical articles were evaluated (196 full articles, 1256 preclinical outcomes from full articles). Main differences were higher frequency of a statement that regulatory requirements were fulfilled and fewer authors did not state on inoculation type (3% vs. 9%) or data variation (20% vs. 26%).

### Development of Quality Parameters over Time

**Fig 3:**
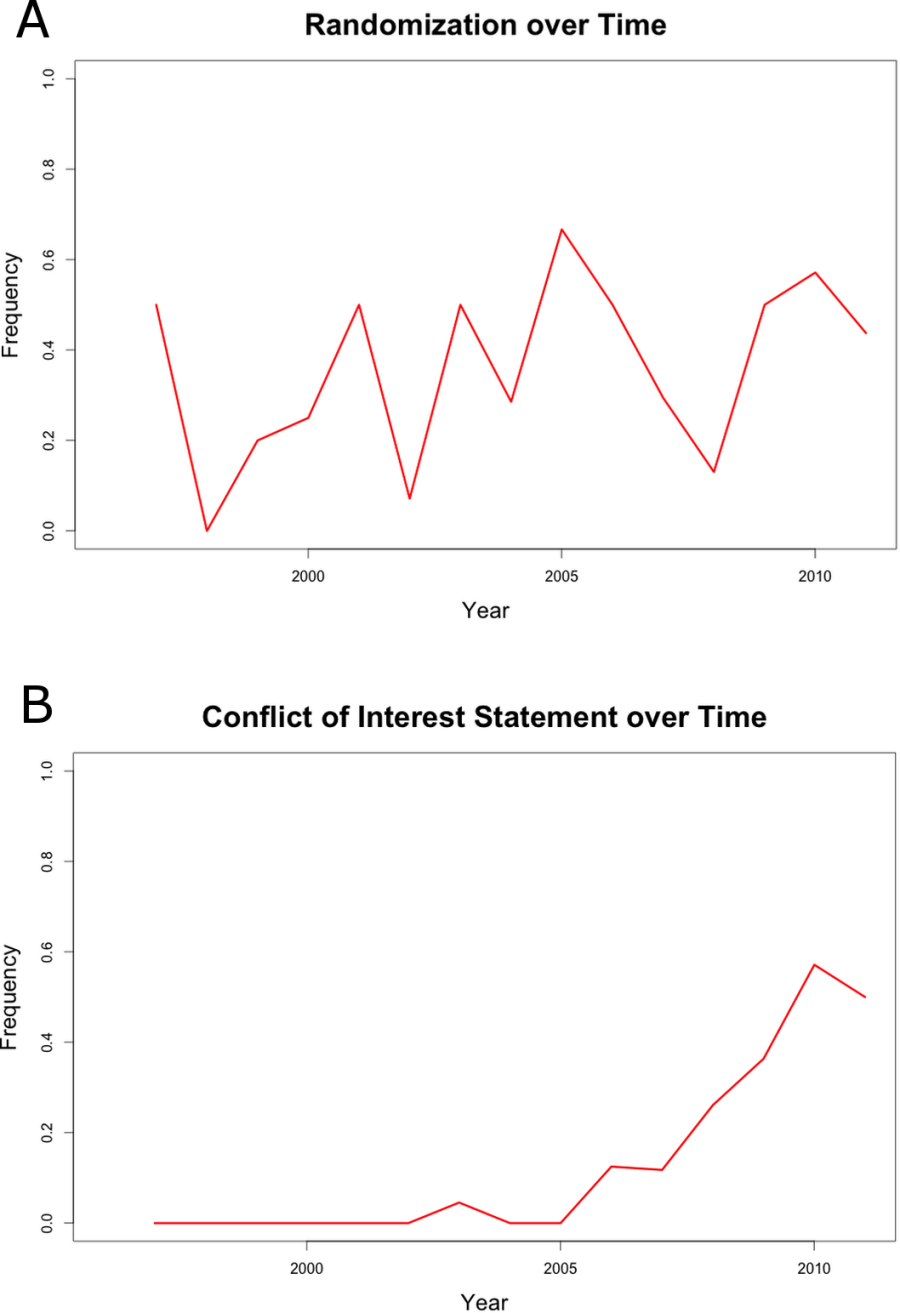
A. Randomization over time. B. Conflict of Interest Statement over time.

By plotting the fulfillment of quality parameters against the year of article publication no trend towards an increase of study quality was registered (parameter “Randomization” is illustrated representatively, Fig 3A). The only exception to this result is the parameter “Conflict of Interest Statement”, which started to continuously increase from 2005 (Fig 3B).

### Contingency Tables

Contingency table and corresponding chi-squared testing identified one experimental parameter with a clear impact on outcome distribution (chi^2^ Test: P<0.001; Tables 4 and 5). There is a clear distribution discrepancy of outcome, depending on the function of investigated intervention. If a drug (candidate) tested was the main focus of the study, positive outcome is favored, whereas negative outcome is more likely, if a drug was tested as a comparison intervention.

**Table 4:** Contingency table of absolute outcome distribution within interventions in main focus of a study or comparison interventions.

	Main Focus	Comparison Intervention	Total
**Outcome 1–3**	180	5	185
**Outcome 4a**	344	29	373
**Outcome 4b**	290	43	333
**Total**	814	77	891

Absolut distribution of positive (1–3), rather neutral (4a) and negative (4b) outcome within the study parameter “function of intervention”. Chi^2^-Test, P<0.001.

**Table 5:** Contingency table of relative outcome distribution within interventions in main focus of a study or comparison interventions.

	Main Focus (%)	Comparison Intervention (%)	Total
**Outcome 1–3**	22	6	28
**Outcome 4a**	42	38	80
**Outcome 4b**	36	56	92
**Total**	100	100	200

Relative distribution of positive (1–3), rather neutral (4a) and negative (4b) outcome within the study parameter “function of intervention”.

The result showed very similar results when only studies investigating Sutent were included, though the Chi^2^-Test was not significant (n = 96, Table 6).

**Table 6:** Contingency table of relative outcome distribution with Sutent in main focus of a study or comparison interventions.

	Sutent Main Focus (%)	Sutent Comparison Intervention (%)	Total
**Outcome 1–3**	29	10	39
**Outcome 4a**	36	33	69
**Outcome 4b**	35	57	92
**Total**	100	100	200

Relative distribution of positive (1–3), rather neutral (4a) and negative (4b) outcome within the study parameter “function of intervention” for experiments investigating Sutent (n = 96). Chi^2^-Test, P = 0.17.

**Fig 4:**
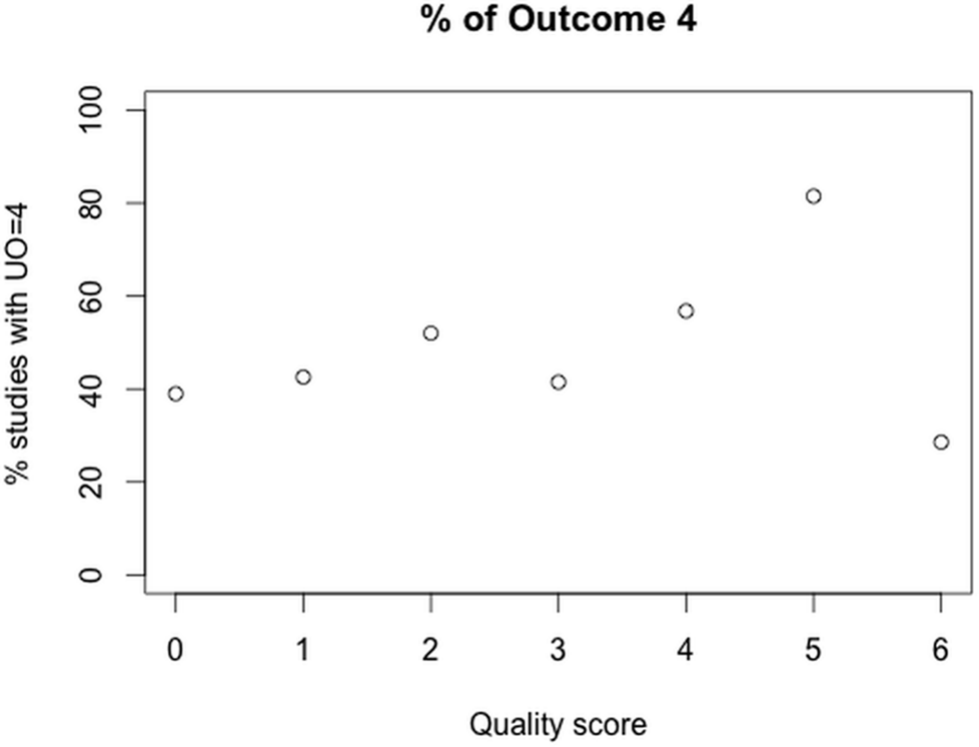
Frequency of experimental outcome 4 (progression) for studies with the same quality score. n = 891.

Chi^2^-Test did not reveal a clear connection between experimental quality and outcome. But there is a tendency towards higher frequency of moderate outcome (4a and 4b) for studies with higher quality (Fig 4).

### Cluster Analysis

**Fig 5:**
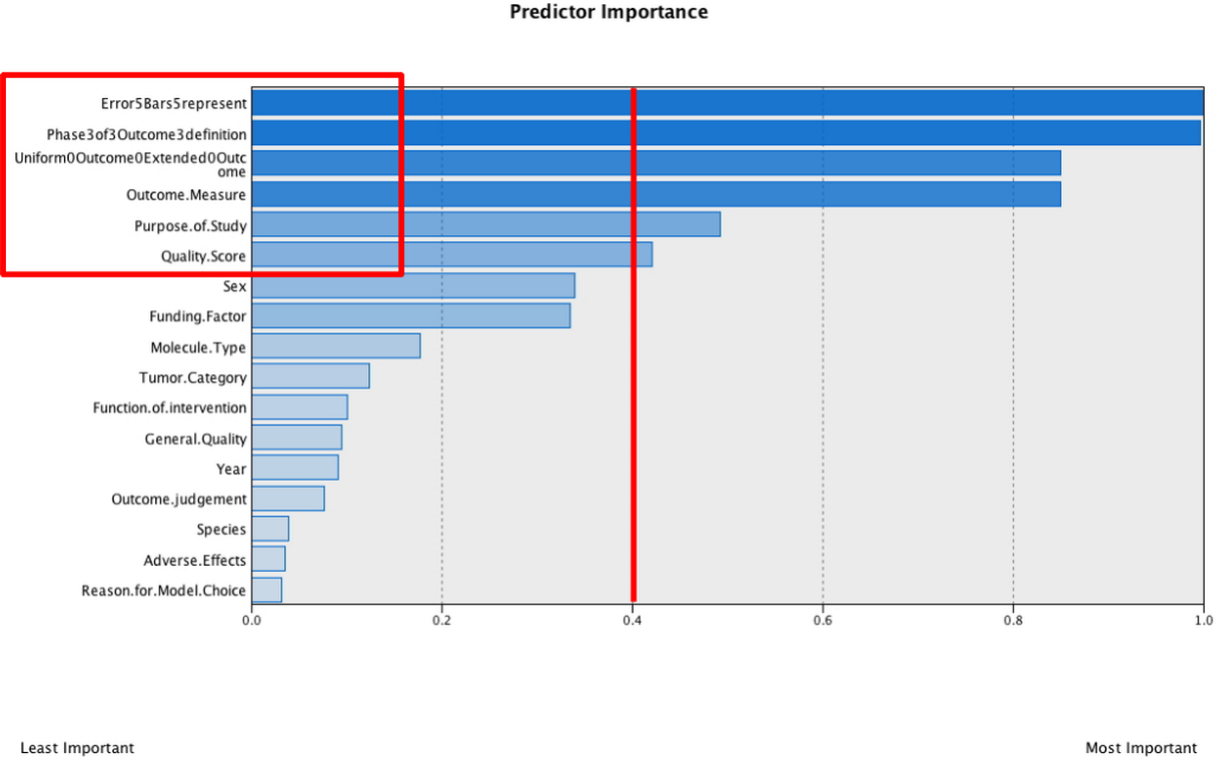
SPSS output predictor importance. Cutoff level = 0.4.

Cluster analysis was performed to identify subgroups of specific combinations of variables within experimental parameters, defining experimental setups that are related in some way. In the first step, a hierarchic cluster analysis was performed to estimate a reasonable number of clusters within the dataset. This preliminary hierarchic cluster analysis of the data set identified four clusters. This number was therefore used as a fixed cluster input for the two-step cluster analysis in SPSS. Input variables for the analysis were categorical (n = 18). Predictor importance output is illustrated in Fig 5. As a cutoff level, 0.4 was chosen.

The analysis was repeated with the six categorical variables within the cutoff level. The second analysis did not lead to further restriction of included variables. The four identified clusters can be described as presented in Table 7.

**Table 7:** Variable distribution within the four identified clusters.

Nr	n	Outcome Measure	Uniform Outcome	Phase of Outcome Definition	Quality Score	Error Bar	Study Purpose
1	460	Tumor Size / Blood Vessel Formation	All outcomes more or less equal	During treatment	2 and 3	SEM / SD / complete data range	Pre-clinical / NA
2	427	Tumor Size / Metastases	3–5 equally	Post-treatment	4–6	SD / complete data range	Pre-clinical
3	312	Survival Analysis	-	-	0–3	-	Screening and Pre-clinical
4	338	NA / Tumor Regression	2>>4b and 5	NA	0>>4	NA / none	Screening

SEM = standard error of the mean; SD = standard deviation; NA = not available. Uniform Outcome: 1 = Cure, 2 = Regression, 3 = Stable Disease, 4a = Moderate Progression, 4b = Progression, 5 = NA.

The clusters can be summarized as follows:

1. Pre-clinical studies: Classic outcome measure, modest quality, equally distributed outcome
2. Pre-clinical studies: Classic outcome measure, good quality, tendency towards unfavorable outcome
3. Screening and Pre-clinical studies: Survival analysis, modest quality, uniform outcome definition not feasible
4. Screening studies: Undefined outcome measure, low quality, trend towards favorable outcome supporting the study hypothesis

## Discussion

The systematic analysis of animal experiments investigating the efficacy of anti-angiogenic drugs revealed rather low to moderate experimental quality with six being the highest quality score (of a maximum of ten). Study design concepts that must be standard in clinical trials (allocation concealment, randomization, blinded assessment of outcome, sample size calculation) were fulfilled in less than 50% of all investigated studies. Generally, the quality scores were rather low with a median of 2. Nevertheless, it has to be born in mind that the inclusion of conference abstracts has contributed to this rather low value.

Data representation and particularly the distribution of data variability within an experiment was only appropriate in 26% of the cases (SD, complete data range, individual data points or statistical methods with representation without variability, i.e. survival analysis). In the rest of the cases data (variability) was either not reported on (31%) or SEM was listed or illustrated (43%). Particularly the SEM is not the appropriate measure for data variability within a single experiment, as it represents the variability of data mean values when experiments are performed repeatedly under the same conditions.

Total number of animals used in the investigated studies was not determinable, as many authors did either not state animal numbers or just give vague specifications in their methods section: often found were number ranges (e.g. 6–8 animals per test group). Exact numbers per treatment arm were mostly missing, making it impossible to determine potential attrition rate in the course of the experiment.

Unexpectedly, an improvement in reported study design over time was not detectable. The only parameter with a clear increase over time was the conflict of interest statement. The explanation for this phenomenon is simple, as more and more scientific journals started to explicitly ask for conflict of interest statements in their author’s guidelines. These results support the conclusion that clear guidance with negative consequences upon non-fulfillment from sides of journal editors or official authorities (i.e. article is not getting published) are necessary to enforce certain concepts. This is remarkable as it has been widely discussed in literature, what the negative impacts of low study quality can be [22].

Neglect of blinded assessment of outcome might be the reason that drug candidates that were in the main focus of the study led to higher frequency of desirable experimental outcome than drugs that were investigated as comparison interventions with the main focus of the study being the aim to show that a different drug candidate can perform better. A limitation of this analysis is the unbalanced distribution of experiment numbers between the two categories “main focus” and “comparison intervention”.

The cluster analysis defined four clusters of a similar number of experiments per cluster. Two of those clusters are clearly distinct from the rest, defined as mainly screening experiments (cluster 4) or survival studies (cluster 3), for which it was not feasible to define an outcome score according to RECIST.

The good news of the cluster analysis is that the cluster with mainly pre-clinical experiments (cluster 2) showed clearly the highest study quality, the way in which data was presented was appropriate with either standard deviations or complete data ranges and outcome was determined post-treatment frequently. Nevertheless, the cluster analysis confirms the trend that high study quality leads to higher rate of unfavorable outcome (4a and 4b) compared to the clusters with rather modest (cluster 1) or low study quality (cluster 4), where outcome was either favorable (1–3) or equally distributed. In reverse, this means that low study quality leads to an overestimation of outcome by trend. This trend was also confirmed by visual inspection of outcome vs. quality score (Fig 4). There was one outlier for experiments with highest quality score (6) with lowest fraction of outcome score 4. It can be assumed that this finding is not representative, as only three studies reached quality score 6, describing a total of seven anti-angiogenic treatment arms. Apart from this outlier, the finding confirms the findings of Sena et al. for studies in the field of experimental stroke [13].

The limitation of this cluster analysis is that not all experimental outcome was converted to a uniform outcome score and therefore it was difficult to distinguish favorable and unfavorable outcome for all studies. Furthermore, conference abstracts were also included in the analysis, with the logic consequence that study quality was judged rather low, as the experimental details in an abstract are available rather limitedly. It can be expected, though, that the results would not change much, as the distribution of study parameters was only marginally deviating from the one including conference abstracts.

Generally, it can be stated that animal studies in oncology, represented here as the subpopulation of anti-angiogenesis drug development, suffers from similar problems as other fields of research (e.g. experimental stroke or ALS) [13, 23]. Study quality and data representation determined via available information within publications is mostly insufficient, even though many articles in the recent past have shown consequences of poor study quality on outcome and external validity of results [13, 22–30] and even though the ARRIVE guidelines (Animal Research: Reporting of In Vivo Experiments) were propagated by several scientific journals including this one [31]. These guidelines are a helpful tool for scientists even in the planning phase of experiments in order to achieve maximal reliability of results.

It can be concluded that such guidelines have to be enforced with corresponding negative consequences upon non-performance by journal editors, local authorities or financial sponsors.

## Supporting Information

S1 Table Main parameters extracted from individual studies. http://www.collegium.ethz.ch/de/forschungsprojekte/fellowprojekt-reproduzierbarkeit/medikamentenentwicklung-aussagekraft-von-tierversuchen/Extraction Parameters–Quality of Animal Experiments in Anti-Angiogenic Cancer Drug Development. Explanation for numeric factor levels in Table S1 can be found under: http://www.collegium.ethz.ch/de/forschungsprojekte/fellowprojekt-reproduzierbarkeit/medikamentenentwicklung-aussagekraft-von-tierversuchen/Numeric Key of Factor levels for extraction parameters.
(DOC)
